# Can the fNIRS-derived neural biomarker better discriminate mild cognitive impairment than a neuropsychological screening test?

**DOI:** 10.3389/fnagi.2023.1137283

**Published:** 2023-04-11

**Authors:** Jin-Hyuck Park

**Affiliations:** Department of Occupational Therapy, College of Medical Science, Soonchunhyang University, Asan, Republic of Korea

**Keywords:** mild cognitive impairment, functional near-infrared spectroscopy, hemodynamic response, biomarker, discrimination

## Abstract

**Introduction:**

Early detection of mild cognitive impairment (MCI), a pre-clinical stage of Alzheimer’s disease (AD), has been highlighted as it could be beneficial to prevent progression to AD. Although prior studies on MCI screening have been conducted, the optimized detection way remain unclear yet. Recently, the potential of biomarker for MCI has gained a lot of attention due to a relatively low discriminant power of clinical screening tools.

**Methods:**

This study evaluated biomarkers for screening MCI by performing a verbal digit span task (VDST) using functional near-infrared spectroscopy (fNIRS) to measure signals from the prefrontal cortex (PFC) from a group of 84 healthy controls and 52 subjects with MCI. The concentration changes of oxy-hemoglobin (HbO) were explored during the task in subject groups.

**Results:**

Findings revealed that significant reductions in HbO concentration were observed in the PFC in the MCI group. Specially, the mean of HbO (mHbO) in the left PFC showed the highest discriminant power for MCI, which was higher than that of the Korean version of montreal cognitive assessment (MoCA-K) widely used as a screening tool for MCI. Furthermore, the mHbO in the PFC during the VDST was identified to be significantly correlated to the MoCA-K scores.

**Discussion:**

These findings shed new light on the feasibility and superiority of fNIRS-derived neural biomarker for screening MCI.

## 1. Introduction

Mild cognitive impairment (MCI), a pre-clinical stage of Alzheimer’s disease, is characterized by cognitive declines that exceed those of healthy aging (Park, 2020). MCI has been highlighted because of cognitive resilience through cognitive intervention at an early stage of Alzheimer’s disease (Cummings et al., 1998). Accordingly, a variety of neuropsychological screening tools for MCI have been developed and its clinical usefulness has been confirmed (Nasreddine et al., 2005; Park, 2020). Among these assessments, the mini-mental state examination (MMSE) or the montreal cognitive assessment (MoCA), a brief screening tool to distinguish people with MCI from healthy individuals has been widely used in clinical settings (Park, 2020).

Unfortunately, however, these tools require skilled clinicians and introduce subjectivity to their outcomes (Park et al., 2018). In addition, it is known that the MMSE and the MoCA performances could be affected by education levels and ages. Accordingly, several prior studies indicated that the MoCA has a critical limitation in its discriminant power for MCI in spite of its high validity and reliability (Park and Heo, 2017; Park et al., 2018). These findings imply that more objective and powerful screening tools for MCI need to be introduced.

To address these limitations, brain imaging methods such as functional magnetic resonance imaging (fMRI) and positron emission tomography (PET) have been adopted to complement the objective aspect which conventional screening tools have (Beishon et al., 2017; Vermeij et al., 2017; Katzorke et al., 2018). Indeed, prior studies showed that brain atrophy and the change of brain activity captured by fMRI can differentiate MCI from normal aging (Liang et al., 2011; Katzorke et al., 2018). Specifically, dorsolateral prefrontal cortex activity patterns investigated by fMRI during resting state could be one of biomarkers to distinguish MCI (Liang et al., 2011).

Among these methods, functional near-infrared spectroscopy (fNIRS) is a non-invasive method using light within the near infrared range to measure the concentration changes of the oxy-hemoglobin (HbO) and deoxy-hemoglobin (HbR) in brain regions of interests (Zafar and Hong, 2018). fNIRS is portability, non-invasive, relatively low cost, and high temporal resolution compared with fMRI (Ferrari and Quaresima, 2012). Therefore, fNIRS has been actively employed with its advantages over other methods.

A number of studies have examined the feasibility of hemodynamic responses measured by fNIRS to compare normal aging and individuals with MCI (Niu et al., 2013; Beishon et al., 2017; Yap et al., 2017). The findings of these studies consistently indicated that people with MCI exhibit lower brain activity in the prefrontal cortex (PFC) compared to healthy people while performing working memory tasks. Specifically, in a previous study, subjects were asked to perform a verbal span task while brain activities from the PFC were measured. As the result, the mean value of HbO in the PFC was lower in people with MCI than healthy control during cognitive testing (Li et al., 2018). This finding suggests that decreased activity in the PFC during working memory testing could be a biomarker to differentiate MCI from normal aging (Niu et al., 2013; Beishon et al., 2017; Yap et al., 2017; Li et al., 2018). On the other hand, a previous study analyzed hemodynamic responses measured using fNIRS and found that a 5–28 s window had the highest accuracy in detecting MCI (Khan et al., 2022), suggesting the importance of temporal features of fNIRS. Interestingly, previous studies have also reported the resilience of MCI, finding that acupuncture therapy bring the hemodynamic responses of MCI patients closer to those of healthy controls (Ghafoor et al., 2019; Khan et al., 2022).

Although brain signals from the PFC during working memory testing have been identified to play a role in biomarker for MCI, it is unclear yet how sensitively and specifically brain activities discriminate MCI from normal aging as a biomarker compared to conventional screening tools. Therefore, this study was to compare the discriminant power for MCI between the fNIRS-derived neural biomarker and the MoCA. This study hypothesized that the fNIRS-derived neural biomarker would show higher discriminant power than the MoCA as the biomarker is more objective than paper-based outcomes.

## 2. Materials and methods

### 2.1. Subjects

All participants consisted of 84 healthy older adults and 52 older adults with MCI and they were recruited from local senior centers in South Korea. Based on previous studies (Petersen, 2004; Park, 2020), amnestic MCI, one of the subtypes of MCI, was defined as MCI. According to these studies, the inclusion criteria for MCI were defined as follows: (1) a subjective memory complaint, (2) objective memory impairment confirmed by performances on the neuropsychological battery (Seoul Neuropsychological Screening Battery), (3) intact global cognitive function confirmed by the Korean version of mini-mental state examination, (4) independent activities of daily living, and (5) no dementia diagnosis confirmed by a physician. The exclusion criteria were as follows: (1) the presence of psychiatric or neurological disorders and (2) the presence of auditory or visual impairments. No healthy older adults had memory complaints and they all were in normal range of the neuropsychological battery.

All participants were given a detail description of this study before the beginning of the experiment and they provided written informed consent. The experiment of this study was conducted according to the latest Declaration of Helsinki. This study was approved by local Institutional Review Board and registered at Thai Clinical Trials Registry (no. TCTR20220418003).

### 2.2. Apparatus

**Figure 1:**
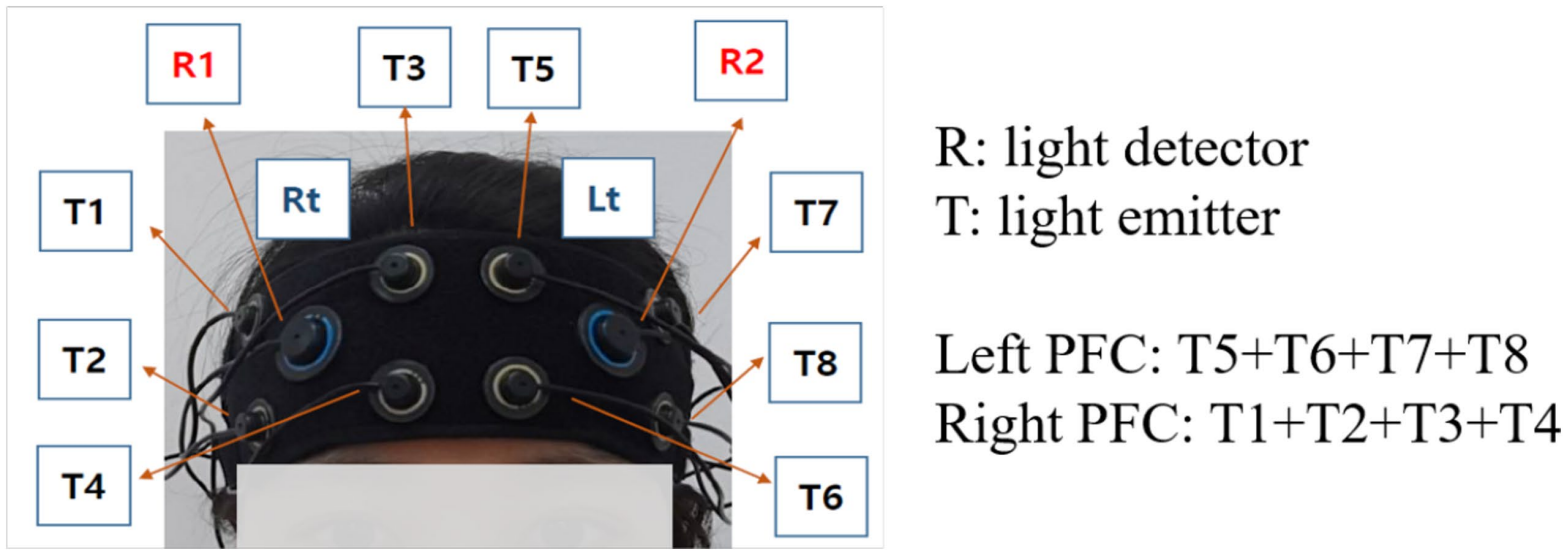
The montage of fNIRS channels.

In this study, a multi-channel fNIRS device (OctaMon, Artinis Medical Systems BV, Elst, The Netherlands) with 8 light emitters and 2 light detectors was used to measure the brain signals at a sampling rate of 10 Hz (Villringer et al., 1993). The wavelengths used for HbO and HbR detection were 760 and 850 nm, respectively. Inter-optode distances were fixed at 3 cm and a total of 8 channels were distributed throughout the frontal cortex regions targeting the left and right dorsolateral PFC (Maidan et al., 2017; Figure 1), according to the international 10–20 electroencephalography (EEG) placement system. All emitters and detectors were mounted to an elastic band, which ensured that 10 optodes made good contact with the participant’s head.

### 2.3. Procedures

All experiments were implemented in a quiet room to reduce environmental disturbance that could affect participant’s performance. Before the beginning of the experiment, detailed information was presented regarding cognitive tasks to participants. In addition, all subjects were instructed to avoid their body movement as much as possible during the experiment.

**Figure 2:**
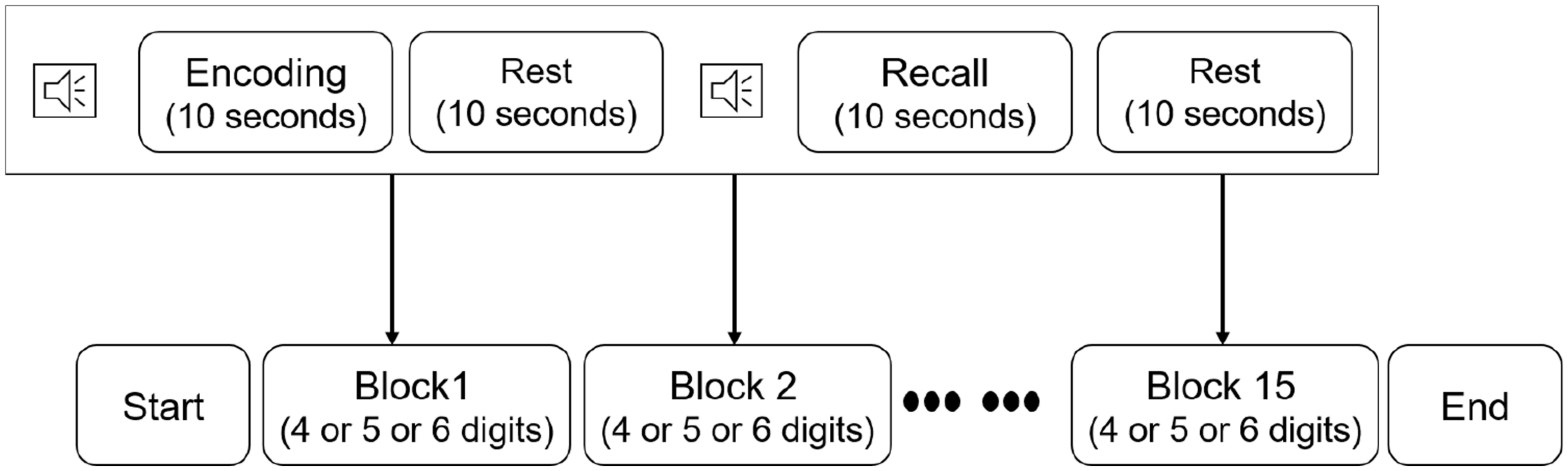
The verbal digit span task design.

Before implementing the verbal digit span task (VDST), participants were seated in a comfortable chair and wore the fNIRS device. Afterwards, participants were instructed to take a rest time with eyes closed for 3 min to stabilize their brain signals. Participants then perform the VDST consisting of 15 individual blocks (Figure 2). Each block started with an encoding period, during which each participant was asked to memorize a number sequence given on a computer screen. Number sequences varied in length from 4 to 6 digits with individual digits ranging from 1 to 9. Subsequently, the participants were asked to verbally recall the number and an experimenter recorded responses for performance evaluation in a recall period. The encoding and recall were followed by a 10 s resting period, during which the participants were instructed to stay relaxed. All sequences in each block were randomly presented to the participants. Practice sessions were given enough to the participants, allowing them to be familiar with this procedure. During each block, hemodynamic signals from the dorsolateral PFC were measured by using the fNIRS device.

After completing the VDST, a 10 min resting period was given to participants. Afterward, participants performed the Korean version of the montreal cognitive assessment (MoCA-K). The MoCA-K consists of seven cognitive domains (visuospatial/executive function, naming, memory, attention, language, abstraction, and orientation). The total of scores ranges from 0 to 30 points, where higher scores indicate better cognitive function. The cut-off score of the MoCA-K for MCI was 23 points, and one point was added if participants had an education level with less than 6 years.

One independent assessor, an occupational therapist with clinical experiences of 4 years, conducted the VDST and the MoCA-K. The assessor was unaware of participant’s characteristics and the purpose of this study.

### 2.4. Data preprocessing

The fNIRS data were collected using OxySoft software (version 3.0.52. Artinis Medical Systems BV, Elst, The Netherlands). This study used HbO signals from each hemisphere (4 channels: right PFC and 4 channels: left PFC) and they were averaged. Each fNIRS channel was visually inspected and channels with large spikes with a standard deviation of 300 μM/mm from the mean which is considered as a noisy were excluded from analysis (Hu et al., 2015).

The fNIRS data were preprocessed by a 4th order Butterworth band-pass filter (cut-off 0.01–0.2 Hz) and an eigenvector-based spatial filtering (Zhang et al., 2005) to reduce artifacts including respiration and cardiac interference. The concentration changes of hemoglobin were computed according to the modified Beer–Lambert Law (Scholkmann et al., 2014). This study focused solely on HbO signals as it is sensitive fNIRS parameter than HbR to cognitive task-associated changes (Villringer et al., 1993).

For each cleaned fNIRS channel, HbO signals of the encoding period in each block were segmented, resulting in 15 trials of HbO signals. Subsequently, all segmented HbO signals were averaged for each channel and each participant.

### 2.5. Data analysis

Mean change of HbO concentration (mHbO) was derived for each of the two groups as suggested by a previous study. In a previous study, mHbO implies the activity of local neurons, which is tied to functional activity and may take more than 10 s to reach a significant activation level (Kato et al., 2017). Therefore, this study calculated the mHbO from the PFC using a 3–12 s window.

### 2.6. Statistical analysis

All data were analyzed by using the SPSS version 22.0. To exam the differences in general characteristics between the healthy group and MCI group, chi-square or independent *t*-test was used. The differences between the healthy group and MCI group in the performance on the VDST, MoCA-K scores, and mHbO were examined using Independent *t*-test. The sensitivity and specificity were evaluated using the receiver-operating characteristics (ROC) curve. The ROC curve is a graphical method for evaluating the performance of binary classifiers such as diagnostic tests. Sensitivity and specificity are commonly used for measures of a classifier’s performance. Also, the Youden index defined was calculated to confirm discriminant powers. The Youden index ranges from 0 to 1, with higher value indicating better discriminant power of a classifier. To examine the predictability, positive predictive value (PPV) and negative predictive value (NPV) were calculated. The PPV indicates that the test is reliable in identifying those who have a disease or condition while the NPV indicates that the test is reliable in ruling out those who do not have a disease or condition. To assess the association between clinical screening tool and hemodynamic signals, Pearson’s correlation coefficients were calculated. All statistical significances were set at *p* < 0.05.

## 3. Results

### 3.1. General characteristics of subjects

The mean age of the healthy group and the MCI group was 73.61 years and 75.35 years, respectively. The percentage of males in each group was 72.3 and 67.7%, respectively. The mean education duration for each group was 6.88 years and 6.00 years, respectively (Table 1). No significant difference in age, sex ration, education periods, and global cognition between the healthy group and MCI group was found (Table 1).

**Table 1:** General characteristic of participants (*N* = 136).

	Healthy group (*n* = 84)	MCI group (*n* = 52)	*t*/*χ*^2^	*p*
Age (year)	73.61 (6.38)	75.35 (5.98)	1.588	0.115
Sex (male/female)	37/47	21/31	0.176	0.723
Education period (year)	6.88 (4.23)	6.00 (4.32)	1.170	0.244
MMSE-K score (point)	26.21 (1.46)	26.02 (1.21)	0.804	0.423

Shown are mean value (standard deviation). MMSE-K, mini-metal state examination.

### 3.2. Behavioral performance and fNIRS analysis

The healthy group outperformed the MCI group in both the MoCA-K and the VDST (*p*’s < 0.001), indicating that the healthy group showed a better global cognition and working memory than the MCI group (Table 2). On the other hand, in brain signals, no noisy channel was observed, and there were significant differences between the healthy group and the MCI group in left, right, and total PFC (*p*’s < 0.001; Table 2). Specifically, the MCI group had a significant decrease in mHbO from both the left and right PFC regions during the VDST compared to the healthy group.

**Table 2:** Behavioral performance and fNIRS analysis (*N* = 136).

		Healthy group (*n* = 84)	MCI group (*n* = 52)	*t*
MoCA-K score (point)		25.86 (1.74)	22.44 (1.95)	10.595^***^
VDST performance (point)		14.45 (0.75)	13.35 (1.10)	6.965^***^
mHbO (μM/mm)	Left PFC	0.977 (0.244)	0.591 (0.163)	10.100^***^
	Right PFC	0.895 (0.222)	0.655 (0.138)	7.495^***^
	Total PFC	0.936 (0.220)	0.623 (0.148)	9.052^***^

^***^p < 0.001. Shown are mean value (standard deviation). mHbO, mean of oxy-hemoglobin; MoCA-K, Korean version of the montreal cognitive assessment; PFC, prefrontal cortex; VDST, verbal digit span task.

### 3.3. Sensitivity, specificity, and discriminant power

**Figure 3:**
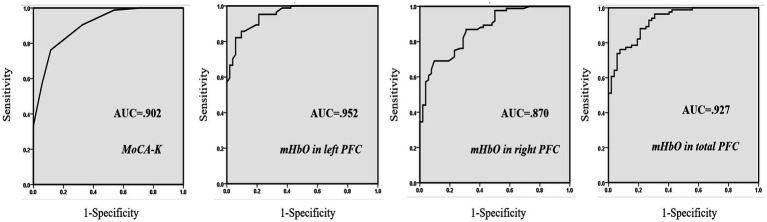
ROC curves of three predictors. Greater AUC values indicate higher power in discriminating MCI from healthy subjects.

For dissociating MCI from healthy controls, the MoCA-K had 76.2% sensitivity and 88.5% specificity with a cut-off score of 24.5 points. On the other hand, a cut-off score of 0.798 for mHbO in the left PFC yielded maximum sensitivity (82.1%) and specificity (94.2%), whereas mHbO in the right PFC showed 69.0% sensitivity and 90.0% specificity. mHbO in the total PFC showed 76.2% sensitivity and 92.3% specificity (Table 3; Figure 3). These findings suggest that hemodynamic signals in the left PFC or the total PFC during cognitive testing can better discriminate MCI compared to the clinical screening tool.

**Table 3:** Sensitivity and specificity of MCI detection *via* machine learning algorithms compared with original tests (*N* = 136).

		Sensitivity	Specificity	Youden index	AUC	Cut-off
MoCA-K score (point)		0.762	0.885	0.647	0.902^***^	24.50
mHbO (μM/mm)	Left PFC	0.821	0.942	0.764	0.952^***^	0.798
	Right PFC	0.690	0.900	0.594	0.870^***^	0.802
	Total PFC	0.762	0.923	0.685	0.927^***^	1.594

^***^p < 0.001. AUC, area under the curve; mHbO, mean of oxy-hemoglobin; MoCA-K, Korean version of the montreal cognitive assessment; PFC, prefrontal cortex.

With respect to the predictability of MCI, mHbO in the left PFC had the highest PPV and the NPV (Table 4). The PPV and NPV of the MoCA-K were higher than those of mHbO in the right PFC. On the other hand, The PPV and NPV of the mHbO in the total PFC were higher those of the MoCA-K (Table 4). These results implied that hemodynamic signals in the left PFC and the total PFC could be more precise in identifying MCI from healthy aging than the conventional screening tool.

**Table 4:** Predictability of the mHbO value and the MoCA-K to distinguish between healthy controls and people with MCI (*N* = 136).

Characteristics			Classification		Predictability
			Healthy group (*n* = 84)	MCI group (*n* = 52)	
MoCA-K score (point)	Healthy		64	5	PPV: 92.75%
	MCI		20	47	NPV: 70.15%
mHbO (μM/mm)	Left PFC	Healthy	70	3	PPV: 95.90%
		MCI	14	49	NPV: 77.78%
	Right PFC	Healthy	60	5	PPV: 92.31%
		MCI	24	47	NPV: 66.20%
	Total PFC	Healthy	65	4	PPV: 94.20%
		MCI	19	48	NPV: 71,64%

PPV, true positive/(true positive + false positive); NPV, true negative/(false negative + true negative); mHbO, mean of oxy-hemoglobin; MoCA-K, Korean version of the montreal cognitive assessment; NPV, negative predictive value; PFC, prefrontal cortex; PPV, positive predictive value.

### 3.4. Correlation between the MoCA-K and the hemodynamic signals

The MoCA-K was found to be significantly correlated with mHbO in left, right, and total PFC (left: *r* = 0.500, *p* < 0.001; right: *r* = 0.378, *p* < 0.001; total: *r* = 0.456, *p* < 0.001; Table 5). This finding suggested that hemodynamic signals are associated with cognitive function.

**Table 5:** Correlation the MoCA-K with hemodynamic signals.

Characteristics	mHbO (μM/mm)		
	Left PFC	Right PFC	Total PFC
MoCA-K score (point)	0.500^***^	0.378^***^	0.456^***^

^***^p < 0.001. mHbO, mean of oxy-hemoglobin; MoCA-K, Korean version of the montreal cognitive assessment.

## 4. Discussion

The goal of this study was to evaluate fNIRS-derived signals as a potential biomarker for MCI. For this, this study investigated hemodynamic properties covering healthy subjects and individuals with MCI. By measuring fNIRS signals from the PFC during the VDST, it was found that different patterns of HbO signals between healthy controls and people with MCI. In addition, the fNIRS-derived indexes including mHbO in the left and right PFC were significantly and positively correlated with clinical screening scores. These findings demonstrated the feasibility of utilizing fNIRS as screening and monitoring tools for MCI.

MCI-related atrophy can cause an alteration in the anatomical structures and functional organization of the PFC, affecting the metabolic activity of cortical neuron in the PFC. These changes can be found through brain imaging techniques. Recently, fNIRS studies have tried to mark MCI-linked brain signals in the PFC. In these studies, featured signals in the PFC of people with MCI were identified (Niu et al., 2013; Li et al., 2018). Prior studies consistently reported a significant decrease of HbO in the PFC in patients with MCI during cognitive tasks (Niu et al., 2013; Li et al., 2018). Similarly, in this study, the MCI group showed a significant reduction in mHbO from both left and right PFC regions compared to healthy controls. These findings suggested that the differences in the blood flow response patterns between healthy and pathological aging could be investigated by fNIRS, which is in line with previous studies (Niu et al., 2013; Li et al., 2018). These findings could be attributed by the fact that neural activities in the PFC become inhibited due to its neurodegeneration in people with MCI, resulting in they fail to recruit sufficient PFC resources for cognitive tasks (Niu et al., 2013). Specifically, since the gray matter declines in the PFC appear in individuals with MCI, they could show relative decreases in HbO in the PFC during working memory tasks compared to healthy controls (Park and Bischof, 2022).

Interestingly, mHbO in the left PFC showed the highest Youden index, indicating that mHbO during the working memory task would be the best factor to discriminate MCI from healthy aging. Specifically, although MoCA-K scores have been widely used to distinguish MCI in clinical settings, it showed lower performance in screening MCI than mHbO in the left PFC. The primary factor underlying this finding is based on the characteristics in test items of the MoCA-K. In the MoCA-K, memory items represent 5 out of 30 points although amnestic MCI show an impairment in working and episodic memory, compared to healthy subjects (Park et al., 2018; Park, 2020). In other words, as a memory decline could be a hallmark of amnestic MCI, in order to differentiate amnestic MCI from normal aging, screening tools for amnestic MCI need to be based on an assessment of memory (Park et al., 2018; Park, 2020). Accordingly, mHbO in the left PFC during the VDST, which reflects the cognitive characteristics of amnestic MCI, has the highest discriminant power compared to the MoCA-K. Furthermore, MoCA-K scores might be influenced by subject age and education periods, limiting their discriminant power (Robert et al., 2010). This finding could be a major difference from a previous study that reported significant differences in mHbO in the PFC between MCI and healthy aging (Li et al., 2018). In contrast, this study not only revealed the difference in mHbO in the PFC between MCI and healthy aging, but also demonstrated that mHbO in the left PFC has a greater discriminant power than the existing screening tool for MCI.

Nevertheless, mHbO in the right PFC was identified to be less effective in screening MCI than MoCA-K scores. This disparity between the left and right PFC could be attributed by a neural compensatory mechanism (Cabeza et al., 2002). According to this mechanism, a neural pathway especially in the right PFC is additionally recruited to support impaired brain functions (Cabeza et al., 2002). Similar to this mechanism, the difference in mHbO of the right PFC during cognitive tasks between the healthy group and MCI group was smaller than that in the left PFC, which suggests that the right PFC is supported by other neural passageways. In other words, the right PFC could be functioning even at the MCI stage. Indeed, a prior study indicated that the right PFC of people with MCI can increase recruitment of working memory (Reuter-Lorenz et al., 2000), supporting the findings of this study. Therefore, the compensation mechanism in the right PFC to close the gap with healthy aging might diminish its discriminant power.

In sum, this study demonstrated distinguishable differences in HbO responses from healthy controls and subjects with MCI by applying the working memory task in conjunction with fNIRS measurement. In particular, the correlation analysis showed significantly positive correlation between MoCA-K scores and mHbO signals in the PFC. These findings present an evidence that the fNIRS-derived indexes hold promise as an alternative tool for screening MCI. Recently, cognitive declines have been detected using a hybrid technique that combines two brain imaging devices to overcome the shortcoming of each modality. In fact, a previous study shown higher accuracy in detecting Alzheimer’s disease when combining electroencephalography and fNIRS than when using either modality alone (Cicalese et al., 2020). Therefore, future research needs to explore which modality is more useful in combination with fNIRS.

One of the main limitation of this study was that only mHbO derived from fNIRS signals was used to classify MCI. Previous studies reported that statistical analysis could not be satisfying to detect MCI (Yang et al., 2019). Although the proposed index in this study showed the highest discriminant power for MCI and was correlated with clinical rating scores, more effort needs be taken to optimize a neural biomarker. Actually, a prior study proposed that an index combined with machine learning techniques could be promising for discriminating MCI in the fNIRS field (Yang et al., 2019). Another issue could be derived from the fNIRS device used in this study. The fNIRS device has a specific channel configuration for the PFC, resulting in that the whole brain could not be investigated. Former studies suggested that people with MCI have a reduced activation not only in the PFC but also in the hippocampus and the parietal lobe (Johnson et al., 2006; Dannhauser et al., 2008). Indeed, a previous study reported that brain networks in MCI patients were characterized compared to healthy controls, which could be an indicator to detect neural biomarkers (Li et al., 2019). Therefore, a broader brain area than the PFC might present the opportunity for measuring better biomarkers. Thirdly, since this study accessed a relatively small number of subjects with MCI, other sub-types of MCI were excluded. To substantiate the present findings, future studies with a larger sample size would be helpful in ensuring the optimized biomarker based on different sub-types of MCI. Finally, the findings of this study can cause overfitting issues as a cross-validation test was not used. To overcome this issue, cross validation is required. However, since the number of data in this study was relatively small, cross validation was not performed. Nevertheless, this study classified MCI using the ROC analysis, which is commonly used in clinical areas. Indeed, a previous study reported that ROC analysis remains a popular and convenient approach to the evaluation of classification rules, particularly when sample sizes are small (Hand and Till, 2001), supporting the appropriateness of ROC analysis with the small sample size.

The purpose of this study was to investigate whether the fNIRS-derived neural biomarker could better discriminate MCI than the neuropsychological screening test. For this, hemodynamic responses in the PFC in conjunction with a VDST were compared between healthy controls and people with MCI. The greater reduction in mHbO concentration was found in people with MCI compared to healthy controls. In addition, mHbO in the left PFC provided the better powerful discriminant power than the neuropsychological screening tool. These findings lead to the conclusion that the fNIRS-derived index could serve as a promising neural biomarker for MCI.

## Data availability statement

The original contributions presented in the study are included in the article/supplementary material, further inquiries can be directed to the corresponding author.

## Ethics statement

The studies involving human participants were reviewed and approved by Soonchunhyang University. The patients/participants provided their written informed consent to participate in this study.

## Author contributions

The author confirms being the sole contributor of this work and has approved it for publication.

## Funding

This research was supported by Basic Science Research Program through the National Research Foundation of Korea (NRF) funded by Ministry of Education (no. 2021R1I1A3041487) and the Soonchunhyang University Research Fund.

## Conflict of interest

The author declares that the research was conducted in the absence of any commercial or financial relationships that could be construed as a potential conflict of interest.

## Publisher’s note

All claims expressed in this article are solely those of the authors and do not necessarily represent those of their affiliated organizations, or those of the publisher, the editors and the reviewers. Any product that may be evaluated in this article, or claim that may be made by its manufacturer, is not guaranteed or endorsed by the publisher.
